# The Effect of Altitude on Phenolic, Antioxidant and Fatty Acid Compositions of Some Turkish Hazelnut (*Coryllus avellana* L.) Cultivars

**DOI:** 10.3390/molecules28135067

**Published:** 2023-06-28

**Authors:** Ersin Gülsoy, Elif Duygu Kaya, Ayşe Türkhan, Menekşe Bulut, Mubin Koyuncu, Emrah Güler, Figen Sayın, Ferhad Muradoğlu

**Affiliations:** 1 Department of Horticulture, Faculty of Agriculture, Igdir University, Igdir 76000, Turkey; 2 Department of Food Engineering, Faculty of Engineering, Igdir University, Igdir 76000, Turkey; elif.duygu.kaya@igdir.edu.tr (E.D.K.); menekse.bulut@igdir.edu.tr (M.B.); fsayin7667@gmail.com (F.S.); 3 Department of Chemistry and Chemical Processing Technologies, Igdir University, Igdir 76000, Turkey; ayse.turkhan@igdir.edu.tr; 4 Department of Horticulture, Faculty of Agriculture, Bolu Abant Izzet Baysal University, Bolu 14030, Turkey; emrahguler6@gmail.com (E.G.); muradogluf@ibu.edu.tr (F.M.)

**Keywords:** SFA, MUFA, PUFA, DPPH, ABTS, oleic acid, linoleic acid, palmitic acid, stearic acid, gallic acid

## Abstract

Turkey is the leading producer and exporter of hazelnuts, producing approximately 64% of global hazelnut production. This research investigated the effects of cultivars and altitude on the phenolic, antioxidant, and fatty acid compositions of five hazelnut cultivars grown at three different altitudes, 100 m, 350 m, and 800 m, in Ordu province, one of the territories that produce the most hazelnuts. The results showed that the cultivar and location significantly affected phenolic compounds, antioxidant activity, and fatty acid (FA) content. The lowest (2.30 mg/kg-Yağlı) and highest (21.11 mg/kg-Kara) gallic acids were obtained at 100 m. The highest total phenolic content and antioxidant activity were found in the nuts grown at 350 m in the Kara and Palaz cultivars, at 100 m in the Yağlı and Sivri cultivars, and at 800 m in the Çakıldak cultivar. Oleic acid was the predominant FA in the cultivars and possessed a diverse trend according to the altitude and cultivar, ranging from 76.04% to 84.80%, increasing with altitude in all cultivars except Çakıldak. Palmitic acid was the predominant saturated FA followed by stearic acid, which significantly varied according to the elevations. This study suggests that the responses of hazelnuts to altitude depend on the cultivar; hence, a proper approach to producing nuts containing more phenolic, fatty acids, and antioxidant activity includes choosing a suitable cultivar for a specific elevation.

## 1. Introduction

Hazelnuts are considered valuable nuts due to their nutritional composition and potential health benefits. Hazelnuts have versatile applications in various food products and culinary preparations. They are commonly used in the production of pastries, bakery products, ice cream, and dairy products, adding a rich and nutty flavor. Hazelnuts are also popular in confectionery and chocolate products, providing texture and taste [1]. They are incorporated into breakfast cereals, breads, and yogurts, enhancing nutritional value and flavor profiles. Additionally, hazelnuts can be included in soups and salads to add crunch and depth of flavor [2]. They are known to contain significant amounts of amino acids, carbohydrates, and unsaturated fatty acids (USFAs), such as oleic acid, linoleic acid, and linolenic acid [3,4]. Additionally, hazelnuts provide essential amino acids [5], vitamins including vitamin E, vitamin B6, vitamin B2, and folic acid [6,7], as well as minerals such as magnesium, copper, potassium, selenium, and phosphorus [8,9]. Phytosterols [10], antioxidants, and phenolic compounds are also present in hazelnuts, contributing to their overall nutritional quality and potential health-promoting effects [11].

Anatolia is recognized as the homeland of hazelnuts, hosting the natural distribution of valuable wild species and serving as the origin of cultural varieties [12]. Turkey, with its favorable climate and suitable soil conditions, is renowned for producing high-quality hazelnut varieties [13]. Hazelnut cultivation in Turkey is primarily concentrated between latitudes 40–41° and longitudes 37–42°. The coastal regions along the Black Sea offer particularly favorable ecological conditions for hazelnut production within this geographical range [14]. Turkey holds the leading position in hazelnut production, contributing to 62% of the global production with an annual output of approximately 665,000 tons. Following Turkey, other significant hazelnut producers include Italy (140,560 tons), the USA (64,410 tons), and Azerbaijan (49,465 tons) [15].

The quality and chemical composition of hazelnuts’ fruits are influenced by various factors including climate conditions, variety, location, agricultural practices, and microbial infection [16,17,18,19]. Recent studies have specifically examined the effects of high altitudes on the phytochemical and nutritional composition of fruits [20,21]. High-altitude environments, characterized by increased UV radiation and lower temperatures, have been found to stimulate the production of phenolic compounds and antioxidants in certain fruits [22]. Moreover, previous research has indicated that altitude [23] and geographical origin can impact the fatty acid composition of specific hazelnut varieties [24].

While some studies have examined the influence of altitude and geographic locations on the fatty acid composition of hazelnuts in Turkey [13,24], there is a lack of research investigating the effect of altitude on phenolic and antioxidant content. Consequently, this study aimed to address this research gap by investigating the impact of altitude on the fatty acid composition as well as the phenolic and antioxidant content of five commonly cultivated hazelnut cultivars grown at different altitudes.

## 2. Results

### 2.1. Content of Polyphenols

**Figure 1:**
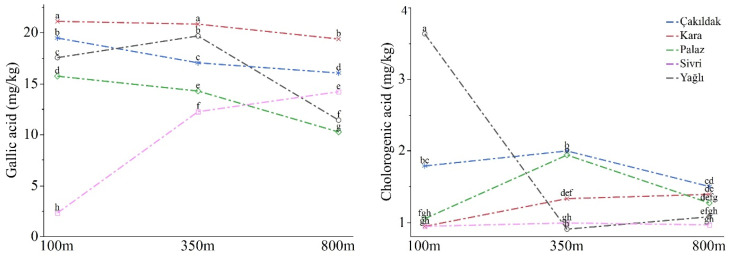
Gallic and chlorogenic acids content fluctuations according to the elevations. Different letters at the top of the lines indicate significant differences for cultivar × altitude interaction according to Tukey’s HSD (*p* ≤ 0.05).

The gallic acid content of hazelnuts was significantly influenced by all sources of variance, including cultivar, altitude, and their interaction (*p* ≤ 0.001). Among the different cultivars, Kara had the highest mean gallic acid content (20.44 mg/kg), followed by Çakıldak (17.52 mg/kg). In contrast, the Yağlı cultivar had a significantly lower gallic acid content, with a mean of 9.58 mg/kg. Hazelnuts grown at an altitude of 350 m exhibited the highest mean gallic acid content (16.82 mg/kg), followed by 100 m (15.23 mg/kg) and 800 m (14.26 mg/kg). The gallic acid content of the cultivars varied significantly with changes in altitude. Specifically, the Yağlı cultivar had the lowest gallic acid content at 100 m elevation, with a mean of 2.30 mg/kg, while the other cultivars showed a range of values similar to the Sivri cultivar. The Çakıldak and Kara cultivars exhibited more consistent gallic acid content across different altitudes, while the other cultivars showed more than 50% variation in gallic acid content due to changes in altitude (Figure 1, Table S1).

The chlorogenic acid content of hazelnuts was significantly influenced by cultivar, elevation, and their interaction (*p* ≤ 0.001). The Sivri cultivar contained the highest mean chlorogenic acid content (1.87 mg/kg), which was not significantly different from that of the Çakıldak cultivar (1.76 mg/kg). The Yağlı cultivar retained the lowest chlorogenic acid content (0.97 mg/kg). The chlorogenic acid content gradually decreased from 1.67 mg/kg at 100 m elevation to 1.43 mg/kg at 350 m elevation and 1.24 mg/kg at 800 m elevation. The Çakıldak and Kara cultivars exhibited relatively stable chlorogenic acid contents across different altitudes, while the Yağlı cultivar exhibited minimal fluctuation of only 5%. In contrast, the Sivri cultivar displayed the highest chlorogenic acid content among the cultivars and delivered nearly four-fold higher chlorogenic acid content at 100 m elevation (3.63 mg/kg) compared to 350 m and 800 m elevations (0.90 mg/kg and 1.08 mg/kg, respectively) (Figure 1, Table S1).

The content of trans-ferulic acid in hazelnuts was significantly influenced by the cultivar, altitude, and their interaction. However, the difference among cultivars was more prominent than the effect of elevation. The trans-ferulic acid content ranged from 1.28 mg/kg (Yağlı) to 2.02 mg/kg (Kara) among the cultivars, while the mean trans-ferulic acid content across the different altitudes varied between 1.63 mg/kg (350 m) and 1.84 mg/kg (100 m). The effect of elevation on the trans-ferulic acid content of hazelnut cultivars was variable. The trans-ferulic acid content of the Yağlı and Sivri cultivars was not affected by elevation. However, the Çakıldak cultivar exhibited significantly different trans-ferulic acid content at each elevation (Table S1).

### 2.2. Total Phenol Content (TPC)

**Figure 2:**
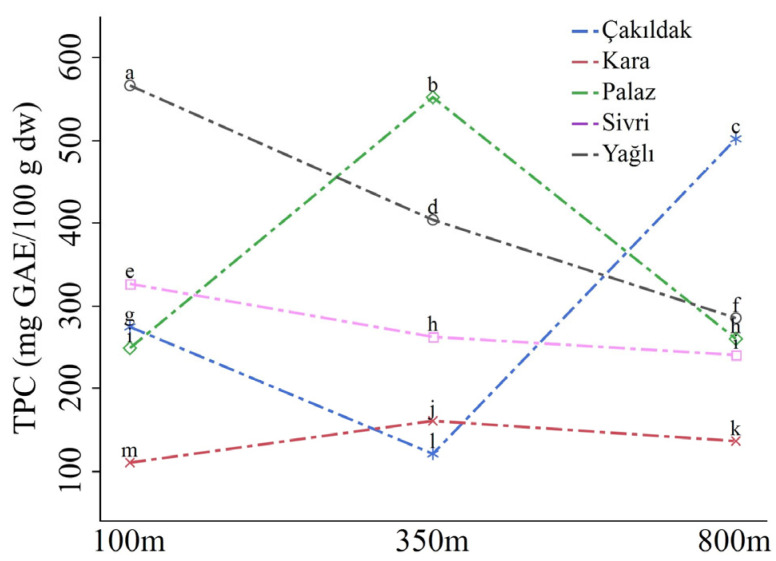
Total phenolic contents of cultivars according to the altitudes. Different letters at the top of the lines indicate significant differences for cultivar × altitude interaction according to Tukey’s HSD (*p* ≤ 0.05).

The total phenolic content (TPC) of hazelnuts significantly varied among cultivars, elevations, and their interaction (*p* ≤ 0.001). The divergence in TPC among cultivars was notable, while the effect of altitude had a relatively limited impact on the mean values. All cultivars demonstrated significant differences in TPC, with the Kara cultivar containing the lowest TPC (mean of 134.97 mg GAE/100 g dw) and the Sivri cultivar containing the highest content of 418.11 mg GAE/100 g dw. Among the cultivars, Çakıldak exhibited the highest TPC at 800 m altitude, while the Kara and Palaz cultivars owned the highest TPC at 350 m. The Yağlı and Sivri cultivars included the highest TPC at 100 m, and the content gradually decreased with increasing elevations (Figure 2, Table S1).

### 2.3. DPPH and ABTS•+ Free Radical Scavenging Activity

**Figure 3:**
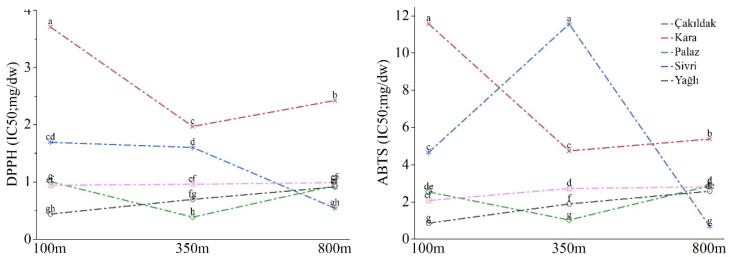
The DPPH and ABTS•+ activity of the cultivars according to orchards’ elevations. Different letters at the top of the lines indicate significant differences for cultivar × altitude interaction according to Tukey’s HSD (*p* ≤ 0.05).

DPPH (2,2-Diphenyl-1-picrylhydrazylradical) and ABTS•+ 2,2-Azino-bis(3-ethylbenzothiazoline-6-sulfonic acid) scavenging activity were significantly affected by cultivar, elevation, and interaction (*p* ≤ 0.001). The Kara cultivar displayed the highest scavenging activities, with means of 7.21 mg/dw for ABTS•+ and 1.76 mg/dw for DPPH, while the Sivri cultivar exhibited the lowest activities, with means of 2.70 mg/dw for ABTS•+ and 0.68 mg/dw for DPPH. Hazelnuts obtained from 100 m elevation possessed significantly higher DPPH activity (1.55 mg/dw) compared to those from 350 m (1.12 mg/dw) and 800 m (1.16 mg/dw) elevations. However, there was a slight and insignificant difference in ABTS•+ scavenging between 100 m (4.32 mg/dw) and 350 m (4.37 mg/dw) elevations, while both were considerably higher than the fruits grown at 800 m (2.85 mg/dw). The ABTS•+ and DPPH activities of the Sivri and Yağlı cultivars increased progressively with elevation, while the other cultivars exhibited significant variations at different altitudes. The Kara cultivar revealed the highest DPPH activity at all altitudes, while the Çakıldak cultivar displayed the highest ABTS•+ activity at 350 m (Figure 3, Table S1).

### 2.4. Fatty Acids (FAs) Fluctuation

**Figure 4:**
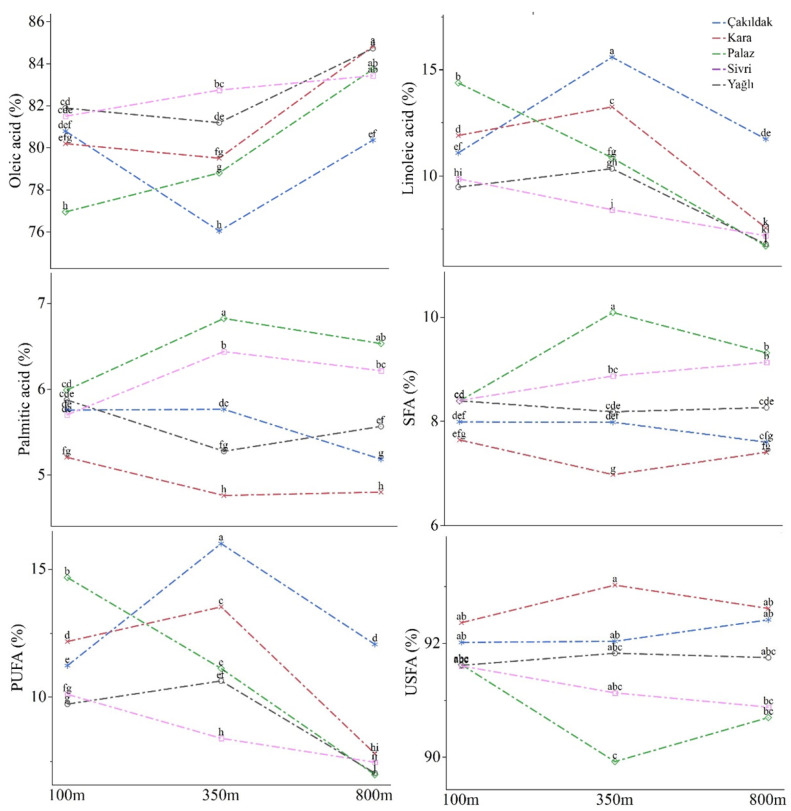
Variations in oleic acid, linoleic acid, palmitic acid, SFA, PUFA, and USFA of hazelnut cultivars according to the altitudes. Different letters at the top of the lines indicate significant differences for cultivar × altitude interaction according to Tukey’s HSD (*p* ≤ 0.05).

Oleic acid was the most abundant fatty acid in the hazelnut cultivars, accounting for approximately 80% of the total fatty acids. Linoleic acid was the second, ranging from 8.47% in the Yağlı cultivar to 12.79% in the Çakıldak cultivar. The other fatty acids detected in the cultivars’ oil were palmitic acid, stearic acid, gamma-linolenic acid, and arachidic acid in order of abundance. These six fatty acids together constituted more than 99% of the total fatty acids in the samples. Oleic acid, linoleic acid, palmitic acid, and stearic acid were significantly influenced by all sources of variance (*p* ≤ 0.01), while arachidic acid was affected by elevation and the interaction between the cultivar and elevation (*p* ≤ 0.05). Gamma-linolenic acid, on the other hand, was not affected by elevation but significantly varied among cultivars and the interactive effect (Figure 4, Table S2).

More than 90% of the FAs were USFAs in the cultivars and varied within the means of 90.74% (Palaz) and 92.66% (Kara). USFAs were mainly comprised of MUFAs (monounsaturated fatty acids) that ranged from 79.06% (Çakıldak) to 82.59% (Sivri). All saturated and unsaturated FAs were significantly diverged by the cultivar, elevation, and their interaction (*p* ≤ 0.01), except for USFAs, which were only affected by the cultivar. The fluctuations in SFAs and USFAs among the cultivars at different elevations were relatively limited. No significant variations were observed among cultivars at different altitudes in terms of USFAs, while SFAs in Palaz and Yağlı were affected. Both MUFAs and polyunsaturated fatty acids (PUFAs) displayed variations at diverse elevations for all cultivars (Figure 4, Table S2).

### 2.5. Statistical Approaches

**Figure 5:**
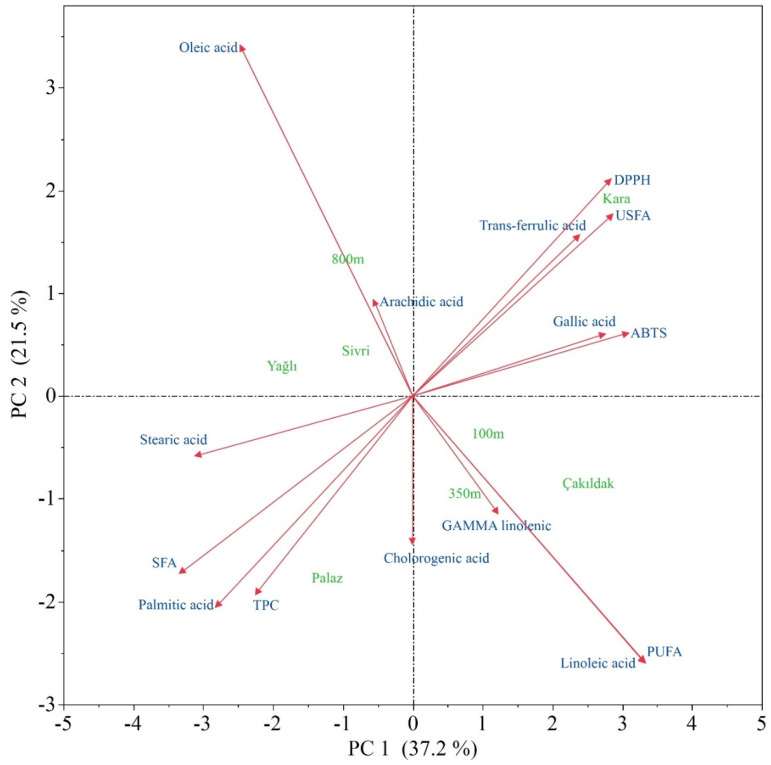
Biplot PCA analysis illustration for the interrelations among the cultivars, altitude, and studied traits. In the biplot, the green-colored variables represent the factors—altitudes and cultivars—while the blue colors represent the studied traits.

The PCA (principal component analysis) showed that the Kara cultivar was characterized by high USFA, trans-ferulic acid contents, and DPPH scavenging activity while possessing low SFA, TPC, and palmitic acid content, which were abundant in the Palaz. The 100 and 350 m elevations were prominent in terms of gamma linolenic acid, linoleic acid, and PUFA, while 800 m was characterized by high arachidic and oleic acids. The Sivri and Yağlı cultivars were close to each other, while the other cultivars were distinct individuals (Figure 5).

**Figure 6:**
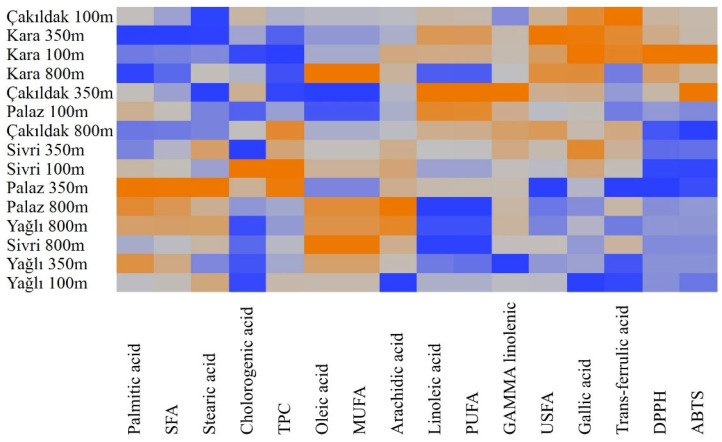
The heatmap analysis illustrates the distribution of nuts of different cultivars grown at different altitudes. The color scale from blue to orange indicates values from low to high.

The heatmap analysis divided cultivars into two main clusters and two subclusters for each. Nuts of the Çakıldak cultivar grown at 100 m altitude were grouped with the nuts of the Kara cultivar from all elevations characterized by relatively low SFA, TPC, palmitic acid, stearic acid, and chlorogenic acid while constituting high USFA, gallic acid, and trans-ferulic acid as well as DPPH and ABTS•+ scavenging activities. Contrarily, the Sivri, Palaz, and Yağlı cultivars were grouped by their high contents of MUFA, SFA, palmitic acid, and oleic acid with low constituents of gallic acid, DPPH, PUFA, linolenic acid, and ABTS. The biochemical changes caused by the altitudes are presented in Figure 6.

## 3. Discussion

The studies mentioned by Göncüoğlu-Tas and Gökmen [25] and others have highlighted the presence of phenolic compounds, such as gallic acid, chlorogenic acid, and ferulic acid, in hazelnuts. These phenolic compounds contribute to the antioxidant activity and health benefits of hazelnuts [26,27]. The reported values of gallic acid, chlorogenic acid, and ferulic acid in hazelnuts vary among different cultivars and methods used for analysis. Temperature variations during hazelnut formation have been found to impact the phytochemical composition of hazelnuts [28,29]. Higher temperatures have been associated with increased total phenolic content (TPC) and antioxidant activity, while colder temperatures have shown a decrease in these parameters [30]. Additionally, environmental factors such as light exposure and UV radiation can influence hazelnut phytochemistry. Hazelnuts exposed to full sunlight have been reported to have higher TPC and antioxidant activity compared to those grown in the shade [31]. UV radiation has also been shown to enhance TPC, flavonoids, and antioxidant capacity of hazelnuts [30]. However, the fatty acid composition of hazelnuts does not appear to be significantly affected by UV radiation [32]. High-altitude environments, characterized by increased UV radiation and lower temperatures, have been suggested to stimulate the production of beneficial plant compounds. UV stress triggers plants’ defense mechanisms, and polyphenols play an active role in the stress response process [33,34]. In the study mentioned, the mean TPC content showed a gradual decrease with increasing elevations, contrary to the general understanding of the impact of UV irradiation and sunlight exposure. However, it is important to note that the response to UV and sunlight exposure may be cultivar-specific, and previous research might have been conducted on UV-tolerant genotypes. Overall, environmental factors, including temperature, light exposure, and UV radiation, can influence the phenolic composition and antioxidant activity of hazelnuts. The specific effects may vary among different hazelnut cultivars and their tolerance to UV radiation. Further research is needed to better understand the interaction between environmental factors and hazelnut phytochemistry to optimize the cultivation and processing of hazelnuts with desired phenolic and antioxidant profiles. The research findings on strawberries, apples, olive oil, avocados, and hazelnuts indicate that altitude can have significant effects on the phytochemical composition of various fruits and their derived products. Total phenolic content and antioxidant capacity decreased gradually with increasing altitudes in strawberries cultivated at different elevations [35]. Similarly, the accumulation of specific health-promoting phytochemicals such as quercetin, epicatechin, and procyanidins in apples was altered by altitude [36]. High-altitude olive fruits were reported to contain higher quantities of heart-healthy fatty acids, particularly oleic acid [37]. On the other hand, the percentage of oleic acid in avocados decreased with increasing elevation, while the percentages of linoleic and palmitoleic acid were enhanced [38]. In the case of hazelnuts, the oleic acid content increased with altitude, while the stearic acid content decreased across all varieties [23]. These findings highlight the fruit-specific nature of the effects of altitude on phytochemical composition. Each cultivar or fruit type may exhibit unique characteristics and responses to changes in elevation. Therefore, it is essential to investigate the relationships between nut traits, including phenolic compounds, fatty acids, and antioxidant capacity, at the variety level to gain a clearer understanding of how altitude influences their composition [17]. In the study, there was a strong correlation between ABTS and DPPH activities, indicating that the antioxidant capacities measured by these two methods were closely related. Both ABTS and DPPH activities were higher in the Kara cultivar compared to other cultivars. However, the altitudes alone did not directly characterize the ABTS and DPPH activities, suggesting that the variations in antioxidant activities observed in the study were primarily influenced by the cultivar rather than the altitudes. It is important to note that this study specifically focused on the antioxidant activities measured by ABTS and DPPH methods, and the results may not reflect the exact antioxidant capacity or the complete antioxidant profile of the hazelnut cultivars.

**Figure 7:**
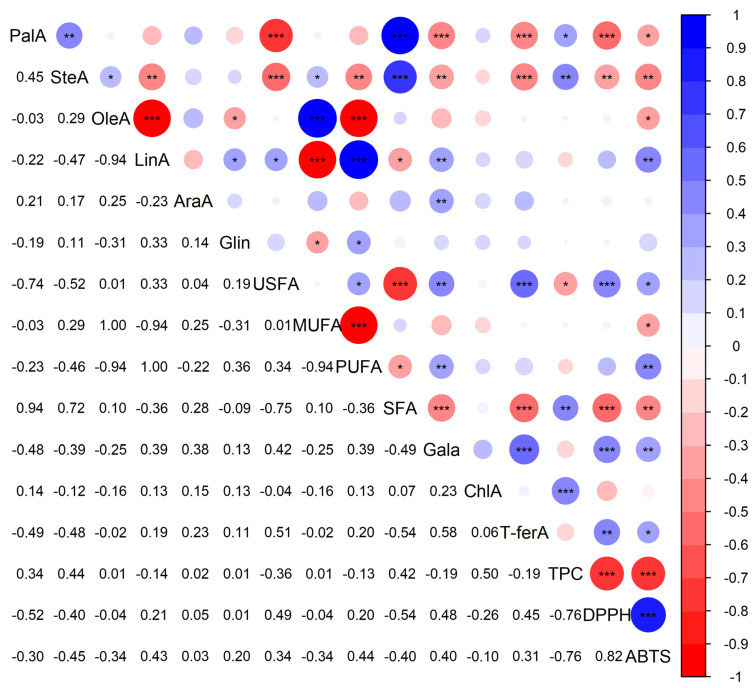
Pearson’s pairwise correlations across the studied characteristics. The upper triangle visually illustrates the redundance of correlations and the *p*-values while the lower triangle includes pairwise correlations. *, **, and *** indicate significant differences at *p* ≤ 0.05, *p* ≤ 0.01, and *p* ≤ 0.001, respectively. PalA; palmitic acid, SteA; stearic acid, OleA; oleic acid; LinA; linoleic acid, Glin; gamma-linolenic acid, USFA; unsaturated fatty acids, MUFA; mono-unsaturated fatty acids, PUFA; polyunsaturated fatty acids, SFA; saturated fatty acids, Gala; gallic acid, ChlA; chlorogenic acid, T-ferA; trans-ferulic acid, TPC; total phenolic content, DPPH; 2,2 Diphenyl 1 picrylhydrazyl scavenging activity, ABTS; 2,2-Azino-bis(3-ethylbenzothiazoline-6-sulfonic acid) scavenging activity.

The analysis of phenolics and fatty acids, without considering varietal and altitudinal distinctions, revealed a lack of strong correlations between these two groups of compounds (Figure 7), suggesting that the phenolic content and fatty acid composition of hazelnuts are influenced by multiple factors, including cultivar and altitude, rather than exhibiting a direct linear relationship. The lack of strong correlations between phenolics and fatty acids indicates that different factors may independently influence their accumulation in hazelnuts. Varietal differences and environmental factors such as altitude can have distinct effects on the phenolic content and fatty acid composition, leading to their non-linear relationship. These findings highlight the complexity of hazelnut chemistry and the importance of considering multiple factors when studying hazelnut composition. Overall, altitude plays a significant role in shaping the phytochemical profiles of fruits and their derived products. Understanding these effects can contribute to optimizing cultivation practices, selecting appropriate varieties for specific altitudes, and promoting the production of fruits with desired nutritional and functional properties.

The findings from previous studies on olives and walnuts indicate that the fatty acid composition, specifically the content of saturated fatty acids (SFAs), is influenced by altitude. For olives, it was observed that higher altitudes were associated with increased SFAs and a lower ratio of polyunsaturated fatty acids (PUFAs) [33,39]. Similarly, in walnuts, the effect of altitude was more pronounced on SFAs compared to unsaturated fatty acids (USFAs), particularly monounsaturated fatty acids (MUFAs) [40]. In the case of hazelnuts, the study by Beyhan et al. [23] revealed that USFAs increased at higher altitudes in certain varieties, namely Karayağlı and Sivri, while SFAs showed a decline. These findings suggest that changes in the fatty acid profile of crops can be influenced by environmental factors and genetic factors such as species and genotype.

## 4. Materials and Methods

### 4.1. Research Sites and Cultivar Choice

This study was performed in three regions of Ordu province, Yemişli, Yeşilyurt, and Turnasuyu, during the 2016 and 2017 growing seasons. Hazelnut fruits were obtained from five cultivars, Çakıldak, Kara, Palaz, Sivri, and Yağlı, which are extensively grown in this region [41].

### 4.2. Fruit Sampling

The fruit samples were gathered from different altitudes: 100 m (Turnasuyu), 350 m (Yemişli), and 800 m (Yeşilyurt). The samples were then dried at 30 °C for 24 h after harvesting, and 100 g nuts for each repetition was stored without shells in a dark room until analysis.

### 4.3. Phenolic Content Analysis

Phenolic compound contents were determined by the method used by the authors of [42] with some minor modifications. A one-gram hazelnut sample was homogenized with a 6000 rpm homogenizer for 5 min after adding 10 mL of 80% methanol and centrifuged at 10,000 rpm for 30 min at 4 °C. The supernatant liquid was passed through a 0.45-micron filter into vials and delivered to the HPLC device equipped with a diode array detector (Agilent 1260, Santa Clara, CA, USA). A 4.6 × 150 mm, 2.7 μm C18 column was used (Poroshell 120 EC-C18), and measurements were carried out between 250 and 450 nm wavelengths. Mobile phase A consisted of 1% phosphoric acid prepared in pure water, and mobile phase B was acetonitrile with a flow rate of 0.8 mL/min. The column temperature was set to 20 °C, and the injection volume was 20 µL. The gradient included 83% mobile phase A and 17% mobile phase B. Different concentrations of gallic acid, chlorogenic acid, epicatechin, rutin, and trans-ferulic acid standards were prepared to create the calibration curve. The standards used in the study were at least 99% pure and obtained from Sigma-Aldrich (Burghausen, Germany).

### 4.4. Total Phenol Content (TPC) Assay

Total phenolic content was determined according to the Folin–Ciocalteu method proposed by the authors of [43]. After adding 250 μL of Folin–Ciocalteu reagent and 50 μL of extract solution to the tube, the total volume was made up to 3 mL with distilled water. After 5 min incubation, 750 μL of 20% (*w*/*v*) Na_2_CO_3_ solution was added, and the tubes were mixed in a vortex. The prepared solution was kept in the dark at room temperature for 90 min, and the absorbance was read at 765 nm in a UV-Vis spectrophotometer (Agilent Cary-60, Santa Clara, CA, USA). The gallic acid standard curve was also created by following the same procedure with concentrations of 50, 100, 150, 200, and 300 µg/mL. Total phenol content was given as gallic acid equivalent (mg GAE/100 g nuts dry weight) using the gallic acid standard curve.

### 4.5. DPPH Free Radical-Scavenging Assay

DPPH free radical scavenging activity was determined by modifying the method of Brand-Williams et al. [44]. Antioxidant activity was defined by measuring the ability of antioxidants to reduce DPPH· radical. A volume of 300 μL of DPPH solution prepared in advance and kept for one (1) night was mixed with 50 μL extract and 2650 μL methanol in a tube and left in the dark for 30 min. Readings were performed at 517 nm in a UV-Vis spectrophotometer (Agilent Cary-60, Santa Clara, CA, USA).

The % DPPH radical scavenging activity was calculated according to the following formula:

DPPH radical scavenging capacity (%) = (A*_control_* − A*_sample_*) × 100/A*_control_*

A*_sample_*: absorbance of the sample;
A*_control_*: absorbance of control.

The inhibition concentration (IC_50_) values for samples were calculated by plotting the inhibition ratios against the regression line obtained from six different sample concentrations and using the regression equation [45].

### 4.6. ABTS Free Radical Scavenging Activity Assay

ABTS•+ radical cation scavenging activity was determined by the method proposed by Bulut et al. [45]. Briefly, a 7 mM ABTS•+ solution containing 2.45 mmol potassium persulfate was prepared. The solution was left in a dark environment for 12–16 h for radical formation at ambient conditions. The dark blue solution was diluted with 20 mM sodium acetate (pH:4.5) until its absorbance was 0.7 ± 0.01 nm at 734 nm wavelength. The time was started by adding hazelnut extracts to the radical solution in the cuvette and the decrease in the absorbance value at the end of the 6th minute was measured at 734 nm in a UV-Vis spectrophotometer (Agilent Cary-60, Santa Clara, CA, USA). The ABTS•+ radicals reduction was calculated by the following formula:

ABTS•+ radical scavenging capacity (%) = (A*_control_* − A*_Sample_*) × 100/A*_control_*

A*_sample_*: absorbance of the sample;
A*_control_*: absorbance of control.

The IC_50_ for samples was also calculated similarly to the DPPH method [45].

### 4.7. Fatty Acids Analysis

A Soxhlet system was utilized to extract oil from finely powdered nuts using hexane (Merck, Darmstadt, Germany). About 0.2 g of the hazelnut oil was transferred to a tube and dissolved in 2 mL hexane. Methyl esters of fatty acids were produced by adding 0.2 mL of a 1 N KOH solution prepared in methanol and violently shaking. The methyl esters were subjected to separation in a gas chromatography device (Agilent 7820-A) equipped with a flame ionization detector (FID) and a capillary column (Restek Rt-2560, 100 m, 0.25 mm, 0.20 µm). Internal standards of FAME 37 Mix (SUPELCO, Bellefonte, PA, USA) were used to identify fatty acids [46].

### 4.8. Statistical Analysis

The study was established in randomized blocks experimental design triplicates on five cultivars and three ocaks (ocak is the cultivation system used for hazelnuts in Turkey) in each replication. The data were subjected to a two-way analysis of variance (ANOVA) by the SPSS 17.0 package program, and the difference between the means was determined by Tukey’s posthoc test at a 95% significance level. The interrelations among studied traits and factors (cultivar and altitude) were clarified by utilizing a principal component analysis (PCA) and a heatmap illustration, with the “ggplot2” package of R Studio software [47].

## 5. Conclusions

This study aimed to investigate the impact of elevation changes on the bioactive compounds in hazelnut cultivation. The results revealed that the alteration of bioactive profiles, including fatty acids and phenolic compounds, in five hazelnut cultivars was not universally influenced by altitude. The Kara cultivar consistently exhibited the highest gallic acid content across all altitudes, exceeding 20 mg/kg. In contrast, the Sivri cultivar displayed a significant increase in gallic acid content from 2.30 mg/kg at 100 m elevation to 14.20 mg/kg at 800 m elevation. Additionally, the total phenolic content of the Çakıldak cultivar varied significantly, possessing a five-fold difference across different altitudes. These findings emphasize the importance of selecting suitable genotypes/cultivars to achieve hazelnuts with desired bioactive compositions. Future research should delve into the molecular mechanisms underlying the changes in phenolic and fatty acid profiles in hazelnuts at different altitudes. The insights gained from this study will aid in selecting appropriate starting materials and contribute to reducing the time required for cultivar selection in future studies.

## Data Availability

Data are available from the corresponding author upon a reasonable demand.

## Supplementary Materials

The following supporting information can be downloaded at: https://www.mdpi.com/article/10.3390/molecules28135067/s1, Table S1: Alteration of the polyphenols, total phenolic content (TPC), and antioxidant activity of hazelnut cultivars according to the altitude; Table S2: The fatty acids composition (%) influenced by cultivar and altitude.

## Sample Availability

Not available.
