# Supplementary material for: The Effect of Altitude on Phenolic, Antioxidant and Fatty Acid Compositions of Some Turkish Hazelnut (Coryllus avellana L.) Cultivars
Source: Molecules. 2023 Jun 28;28(13):5067. doi: 10.3390/molecules28135067 (PMC10343385; doi:10.3390/molecules28135067)
Supplement: Supplementary file 1 [file molecules-28-05067-s001.zip › molecules-2451103-supplementary.pdf]

Table S1. Alteration of the polyphenols, total phenolic content (TPC), and antioxidant activity of hazelnut cultivars according to the altitude

		Cholorogenic acid (mg/kg)	Gallic acid (mg/kg)	Trans- ferrulic acid (mg/kg)	TPC (mg GAE/100 g dw)	DPPH (mg/dw)	ABTS (mg/dw)
Altitude							
100m		1.67±1.07a	15.23±6.96a	1.84±0.54a	304.74±154.38a	1.55±1.19a	4.32±3.95a
350m		1.43±0.48a	16.82±3.34a	1.63±0.44a	299.45±165.39a	1.12±0.61a	4.37±3.92a
800m		1.24±0.22a	14.26±3.40a	1.70±0.25a	284.16±124.15a	1.16±0.68a	2.85±1.55a
Cultivar							
Çakıldak		1.76±0.23ab	17.52±1.55ab	2.00±0.41a	298.46±166.24a	1.28±0.56b	5.61±4.77ab
Kara		1.22±0.22ab	20.44±0.89a	2.02±0.47a	134.97±22.55b	2.70±0.79a	7.21±3.27a
Palaz		1.42±0.41ab	13.42±2.47bc	1.47±0.31bc	353.11±149.19a	0.77±0.30b	2.13±0.85bc
Sivri		1.87±1.33a	16.22±3.72ab	1.84±0.10ab	418.11±122.46a	0.68±0.22b	1.76±0.75c
Yağlı		0.97±0.07b	9.58±5.53c	1.28±0.12c	275.92±38.78ab	0.96±0.05b	2.52±0.36bc
Altitude × Cultivar							
	100m	1.79±0.10bc	19.47±0.22b	2.49±0.08a	273.88±1.05g	1.69±0.07cd	4.64±0.12c
Çakıldak	350m	2.00±0.12b	17.04±0.40c	1.55±0.06def	119.99±1.74l	1.60±0.06d	11.53±0.42a
	800m	1.50±0.01cd	16.04±0.07d	1.97±0.09bc	501.53±1.21c	0.54±0.08gh	0.67±0.06g
	100m	0.94±0.06gh	21.11±0.42a	2.35±0.29a	109.37±8.81m	3.70±0.22a	11.55±0.38a
Kara	350m	1.33±0.06def	20.84±0.26a	2.29±0.09ab	160.03±5.54j	1.97±0.09c	4.72±0.19c
	800m	1.39±0.12de	19.38±0.55b	1.43±0.03efg	135.51±0.89k	2.42±0.16b	5.36±0.22b
	100m	1.05±0.06fgh	15.73±0.40d	1.42±0.04fg	248.15±0.45i	1.00±0.10e	2.52±0.10de
Palaz	350m	1.94±0.15b	14.27±0.19e	1.15±0.10g	551.92±0.39b	0.38±0.06h	1.01±0.10g
	800m	1.27±0.12defg	10.25±0.30g	1.82±0.15cd	259.27±0.77h	0.93±0.11ef	2.85±0.12d
	100m	3.63±0.27a	17.55±0.24c	1.76±0.07cde	566.24±0.83a	0.44±0.07gh	0.84±0.14g
Sivri	350m	0.90±0.06h	19.68±0.14b	1.89±0.09c	403.50±1.46d	0.69±0.04fg	1.88±0.11f
	800m	1.08±0.05efgh	11.42±0.15f	1.86±0.14cd	284.58±0.71f	0.91±0.11ef	2.56±0.07de
	100m	0.94±0.07gh	2.30±0.24h	1.18±0.03g	326.04±0.56e	0.94±0.07ef	2.06±0.11ef
Yağlı	350m	0.99±0.06gh	12.24±0.17f	1.25±0.10fg	261.81±0.63h	0.96±0.04ef	2.71±0.15d
	800m	0.96±0.09gh	14.20±0.26e	1.42±0.05fg	239.90±1.32i	0.99±0.05ef	2.79±0.07d
$F_{Cultivar}$		104.02***	1789.27***	80.03***	12374.39***	611.97***	1490.09***
$F_{Altitude}$		57.62***	290.47***	14.46***	211.7***	86.76***	317.07***
$F_{Cultivar\ x\ Altitude}$		150.49***	548.21***	33.92***	8209.21***	82.54***	898.83***

Different letters in the same column indicate significant differences according to Tukey's HSD test ($p \leq 0.05$). *** indicates sigificance at $p \leq 0.001$.

Table S2. The fatty acids composition (%) influenced by cultivar and altitude.

		Arachidic	Gamma Linolenic	Linoleic	Oleic	Palmitik	Stearic	USFA	SFA	MUFA	PUFA
Altitude											
100m		0.10±0.06a	0.25±0.07a	11.33±1.82a	80.26±1.83b	5.70±0.30a	2.36±0.23a	91.84±0.52a	8.16±0.36a	80.26±1.83b	11.58±1.84a
350m		0.12±0.04a	0.25±0.15a	11.68±2.58a	79.65±2.42b	5.81±0.78a	2.48±0.43a	91.58±1.30a	8.42±1.09a	79.65±2.42b	11.93±2.70a
800m		0.15±0.05a	0.29±0.06a	7.98±1.97b	83.40±1.70a	5.66±0.67a	2.55±0.19a	91.66±0.92a	8.34±0.83a	83.40±1.70a	8.27±2.00b
Cultivar											
Çakıldak		0.12±0.04a	0.30±0.12a	12.79±2.12a	79.06±2.28b	5.57±0.31b	2.17±0.12c	92.15±0.43ab	7.85±0.27cd	79.06±2.28b	13.09±2.21a
Kara		0.13±0.03a	0.27±0.05a	10.89±2.59ab	81.49±2.62ab	4.92±0.24c	2.30±0.19bc	92.66±0.99a	7.34±0.34d	81.49±2.62ab	11.16±2.60ab
Palaz		0.14±0.05a	0.29±0.08a	10.63±3.33ab	79.82±3.04ab	6.45±0.39a	2.68±0.38a	90.74±0.80d	9.26±0.78a	79.82±3.04ab	10.92±3.34ab
Sivri		0.14±0.05a	0.28±0.06a	8.85±1.61b	82.59±1.64a	5.57±0.27b	2.57±0.21ab	91.72±0.38bc	8.28±0.24bc	82.59±1.64a	9.13±1.63b
Yağlı		0.10±0.08a	0.18±0.15a	8.47±1.17b	82.54±0.93a	6.12±0.34a	2.59±0.24ab	91.20±0.64cd	8.80±0.35ab	82.54±0.93a	8.66±1.18b
Altitude × Cultivar											
	100m	0.12±0.05ab	0.16±0.04bc	11.07±0.04ef	80.78±0.35def	5.75±0.15de	2.11±0.12e	92.01±0.42ab	7.99±0.27def	80.78±0.35def	11.23±0.08e
Çakıldak	350m	0.11±0.04ab	0.41±0.06a	15.58±0.37a	76.04±0.19h	5.76±0.17de	2.10±0.10e	92.03±0.54ab	7.98±0.26def	76.04±0.19h	15.99±0.35a
	800m	0.12±0.05ab	0.33±0.05ab	11.72±0.34de	80.35±0.07ef	5.18±0.05g	2.29±0.06de	92.41±0.37ab	7.60±0.05efg	80.35±0.07ef	12.06±0.29d
	100m	0.14±0.01a	0.28±0.03ab	11.89±0.40d	80.19±0.21efg	5.20±0.14fg	2.31±0.16de	92.36±0.60ab	7.64±0.24efg	80.19±0.21efg	12.17±0.41d
Kara	350m	0.11±0.03ab	0.28±0.08ab	13.24±0.31c	79.50±1.47fg	4.76±0.04h	2.11±0.05e	93.02±1.74a	6.97±0.03g	79.50±1.47fg	13.52±0.29c
	800m	0.13±0.03a	0.26±0.06ab	7.54±0.07k	84.80±0.51a	4.80±0.14h	2.48±0.13bcd	92.61±0.47ab	7.40±0.23fg	84.80±0.51a	7.80±0.13hi
	100m	0.11±0.03ab	0.30±0.08ab	14.37±0.19b	76.95±0.08h	5.99±0.06cd	2.29±0.08de	91.62±0.12abc	8.39±0.03cd	76.95±0.08h	14.66±0.16b
Palaz	350m	0.14±0.05a	0.28±0.10ab	10.84±0.17fg	78.80±0.24g	6.82±0.17a	3.13±0.15a	89.92±0.29c	10.08±0.35a	78.80±0.24g	11.12±0.18e
	800m	0.17±0.06a	0.29±0.10ab	6.69±0.12l	83.71±0.45ab	6.53±0.18ab	2.62±0.17bcd	90.69±0.54bc	9.31±0.42b	83.71±0.45ab	6.98±0.18j
	100m	0.14±0.06a	0.27±0.07ab	9.46±0.06i	81.88±0.18cd	5.87±0.11cde	2.37±0.16cde	91.61±0.16abc	8.39±0.29cd	81.88±0.18cd	9.73±0.12g
Sivri	350m	0.14±0.05a	0.31±0.04ab	10.33±0.09gh	81.18±0.38de	5.27±0.09fg	2.77±0.16ab	91.82±0.47abc	8.18±0.28cde	81.18±0.38de	10.63±0.14ef
	800m	0.14±0.05a	0.27±0.07ab	6.77±0.27l	84.70±0.28a	5.56±0.07ef	2.56±0.08bcd	91.74±0.56abc	8.26±0.19cde	84.70±0.28a	7.04±0.28j
	100m	0.00±0.00b	0.26±0.08ab	9.85±0.26hi	81.49±0.51cde	5.70±0.14de	2.70±0.13bc	91.60±0.79abc	8.40±0.04cd	81.49±0.51cde	10.11±0.30fg
Yağlı	350m	0.13±0.04a	0.00±0.00c	8.40±0.15j	82.73±0.32bc	6.44±0.10b	2.30±0.12de	91.12±0.46abc	8.87±0.18bc	82.73±0.32bc	8.40±0.15h
	800m	0.16±0.05a	0.29±0.04ab	7.17±0.16kl	83.41±0.48ab	6.21±0.13bc	2.77±0.08ab	90.87±0.62bc	9.13±0.21b	83.41±0.48ab	7.46±0.18ij
$F_{Cultivar}$		1.64ns	4.79**	522.09***	92.6***	206.59***	28.51***	12.13***	92.66***	92.6***	509.48***
$F_{Altitude}$		4.15*	1.41ns	1199.96***	243.67***	6.27**	9.33***	0.61ns	4.68*	243.67***	1105.42***
$F_{Cultivar\ x\ Altitude}$		2.85*	7.24***	201.05***	34.97***	27.88***	14.22***	1.67ns	12.78***	34.97***	203.73***

Different letters in the same column indicate significant differences according to Tukey's HSD test ($p \leq 0.05$). ns: not significant. *,**, and ** indicates sigificance at $p \leq 0.05$, $p \leq 0.01$, and $p \leq 0.001$, respectively.
